# What Affects the Completion of Ecological Momentary Assessments in Chronic Pain Research? An Individual Patient Data Meta-Analysis

**DOI:** 10.2196/11398

**Published:** 2019-02-05

**Authors:** Masakatsu Ono, Stefan Schneider, Doerte U Junghaenel, Arthur A Stone

**Affiliations:** 1 Center for Self-Report Science Center for Economic and Social Research University of Southern California Los Angeles, CA United States; 2 Department of Psychology University of Southern California Los Angeles, CA United States

**Keywords:** chronic pain, completion rate, compliance rate, ecological momentary assessment, experience sampling, IPD meta-analysis

## Abstract

**Background:**

Ecological momentary assessment (EMA) involves repeated sampling of people’s current experiences in real time in their natural environments, which offers a granular perspective on patients’ experience of pain and other symptoms. However, EMA can be burdensome to patients, and its benefits depend upon patients’ engagement in the assessments.

**Objective:**

The goal of this study was to investigate factors affecting EMA-completion rates among patients with chronic pain.

**Methods:**

This individual patient data meta-analysis was based on 12 EMA datasets that examined patients with chronic noncancer-related pain (n=701). The EMA-completion rates were calculated on a daily basis for each patient. Multilevel models were used to test the following predictors of completion rates at different levels: within-patient factors (days into the study and daily pain level), between-patient factors (age, sex, pain diagnosis, and average pain level per person), and between-study EMA design factors (study duration, sampling density, and survey length).

**Results:**

Across datasets, an EMA-completion rate of 85% was observed. The strongest results were found for the between-patient factor age: Younger respondents reported lower completion rates than older respondents (*P*=.002). One within-patient factor, study day, was associated with completion rates (*P*<.001): over the course of the studies, the completion rates declined. The two abovementioned factors interacted with each other (*P*=.02) in that younger participants showed a more rapid decline in EMA completion over time. In addition, none of the other hypothesized factors including gender, chronic pain diagnoses, pain intensity levels, or measures of study burden showed any significant effects.

**Conclusion:**

Many factors thought to influence the EMA-completion rates in chronic pain studies were not confirmed. However, future EMA research in chronic pain should note that study length and young age can impact the quality of the momentary data and devise strategies to maximize completion rates across different age groups and study days.

## Introduction

Ecological momentary assessment, or EMA [1] (also known as experience sampling), is gaining increasing attention in medical research and research on chronic pain, mainly due to its ability to capture real-time data that reflect the dynamics of patients’ experiences in their natural environment. The methodology involves prompting participants several times per day to answer questions about their current pain and symptoms. This facilitates coverage of people’s experiences under a full range of momentary contexts, circumvents potential recall bias, and enhances the ecological validity of assessments [1,2]. Despite the advantages of EMA, the quality of these data depends on adequate completion rates of the sampling protocol [3]. Specifically, systematic differences in completion patterns according to characteristics of the situation, person, or EMA protocol could lead to input of data that are not missing at random, which could severely bias the results of subsequent analyses [4]. Modern statistical approaches for handling missing EMA data (maximum likelihood estimation or multiple imputation) rely on the assumption that data are missing at random (MAR). For the MAR assumption to be met, variables that are predictive of missing values need to be included in the analysis or imputation model [5]. Therefore, identification of factors facilitating or reducing EMA-completion rates is important.

Although few studies have examined predictors of completion rates in EMA, the available evidence suggests that male sex, engagement in behaviors that draw attention away from participation (eg, drinking alcohol or exercising), and long participation in the protocol may be associated with low completion rates [4,6-9]. However, this research was based on nonpatient samples (eg, college students, healthy adults, and drug or tobacco users). To our knowledge, only two original studies have examined factors associated with EMA-completion rates in patients with chronic pain. Aaron and colleagues [10] examined the completion rates among patients with temporomandibular disorder and found that demographic and medical characteristics were not related to the number of missed EMA surveys; interestingly, participants with high negative mood and high stress tended to have low completion rates. Okifuji et al [11] found no differences in the missing response rates according to patient characteristics (eg, age, pain, and fatigue), but the missing response rates increased over the course of the 30-day EMA study. In addition, Morren and colleagues [12] used meta-analytic procedures to examine the EMA-completion rates in pain research and reported overall high completion rates (average, 83%) among papers that provided the completion rates (36 of 48 studies). In addition, their results suggested that studies with older patients, shorter EMA surveys, participation manuals, alarm functions, and financial incentives had higher average completion rates.

In this study, we used an individual patient data (IPD) meta-analytic approach to investigate predictors of EMA-completion rates in patients with chronic pain [13-15]. There are several advantages to the use of IPD meta-analysis. First, in contrast to traditional meta-analysis, we utilize and synthesize raw data of several EMA studies, which allows examination of predictors that are not reported in the published article or only reported as summary statistics. This avoids potential biases resulting from inferences about individuals made from group-level summary data (“ecological fallacy”). Second, with IPD meta-analysis, it is possible to standardize outcome definitions across studies; this is especially a concern because the conceptualization and quality of reporting of EMA-completion rates varies widely between published studies [16]). Third, IPD allows examination of predictors of completion rates across multiple levels of analysis, including features of the study protocol (study-level predictors), participant-level features (person-level predictors), temporal features (within-person changes over time), and the construct under investigation (ie, pain intensity).

The overall objective of our IPD meta-analysis was to determine the profile of the study level, participant level, and situational features that affect completion rates in EMA research on chronic pain. At a study level, we hypothesized that long EMA questionnaires, long study durations, and high sampling densities may be associated with low completion rates. On the basis of prior related literature, at the participant level, we hypothesized that male sex and young age may be associated with low completion rates [6-8,12]. Further, we hypothesized that completion rates would decrease over time [11] due to participant fatigue or reduced motivation in the later phase of the study. Finally, we hypothesized that chronic pain diagnosis or patients’ pain intensity might predict completion of EMA assessments.

## Methods

### Data Acquisition

The data for this study were sourced from a larger study utilizing secondary data analyses of preexisting EMA datasets for characterizing momentary pain experiences in patients with chronic noncancer-related pain [16]. The inclusion criteria were study sample of at least 30 adult patients (excluding studies with pediatric patients) and administration of a minimum of 3 EMA pain intensity prompts per day for at least 4 days with a fixed- or random-assessment schedule that was assessed via electronic diaries, mobile phones, or interactive voice responses. Studies using paper diaries were excluded because of problems (eg, back-filling and forward-filling) that can undermine the validity of the estimated completion rates [17,18]. Observational studies and clinical trials were included, but clinical trials were limited to no-intervention or baseline assessment periods. EMA pain assessments needed to focus on monitoring momentary pain intensity. Studies that used EMA exclusively as an intervention trigger (eg, just-in-time adaptive interventions [19]) were excluded.

Eligible datasets were identified through a systematic literature search conducted in October 2016 using PubMed and Web of Science databases with the following search terms: ([“Ecological Momentary Assessment” or “Experience Sampling” or “Electronic Diary” or “Electronic Diaries” or “Electronic Interview” or “Electronic Interviews” or “Interactive Voice Response” or “Intensive Diaries” or “Ambulatory Monitoring” or “Ambulatory Assessment”] and “Pain”).

### Analysis Strategy

Completion rates were calculated as the percentage of EMA prompts completed (relative to the number of prompts received) for each person and day of the study. For EMA protocols with a fixed sampling scheme (6 studies), we considered the number of prompts received as the number of prompts specified by the protocol (and reported in respective articles). For EMA protocols with a random-sampling scheme (4 studies), we obtained the specific number of executed prompts from the datasets, because this number could vary across days (this was especially the case when studies allowed the number of momentary prompts to vary according to patients’ waking hours). Given the proportional nature of the completion rates, we tested our models with both the original and arcsine-transformed scores [20]. In this paper, we report the results based on the original completion rates, because the analyses yielded nearly identical results.

To examine changes in completion rates over time, the study day was coded as a within-person (day-level) predictor variable. Data for patient-level predictors of EMA-completion rates—age and sex—were taken directly from the databases. Patients’ chronic pain diagnosis was coded as osteoarthritis, rheumatoid arthritis, fibromyalgia, or other diagnoses. The following features of the EMA protocol were coded as study-level predictors of EMA completion rates: Study duration was coded as the total number of days of the EMA protocol, EMA sampling density was coded as the average number of EMA prompts received per day, and EMA survey length was coded as the number of EMA items presented at each prompt.

We used the momentary pain intensity ratings available in each dataset to examine whether EMA-completion rates were associated with pain intensity at the day, person, or study level. The number of scale points used to measure momentary pain differed across studies (range, 5-101 points), and the pain ratings were converted, so that the ratings were on a 101-point scale. For the conversion, we used the following equation: New rating=100*(original rating+0.5)/(number of scale points). Because momentary pain ratings were not assessed at the time of the missed EMA prompts, the analyses were based on averages of the nonmissing pain ratings, as per a previous analysis [10]. Specifically, study- and person-level averages of all available pain ratings were calculated to examine whether studies or patients who, on an average, reported higher pain levels showed lower (or higher) completion rates. Additionally, daily average pain levels were computed for each patient (and within-person centered) to examine whether day-to-day variations in pain in a given patient were related to daily completion rates.

Our multilevel models subsequently incorporated day-level (Level 1), person-level (Level 2), and study-level (Level 3) predictors of EMA-completion rates. Specifically, a model without predictors (Step 1) was followed by analyses of the day-level predictors, which examined changes in daily completion rates over time (ie, over the course of the EMA sampling protocols; Step 2). Step 3 added patient-level predictors including age, sex, and chronic pain diagnosis. Step 4 added the following study-level predictors: study duration, EMA sampling density, and EMA survey length. In the final step (Step 5), day-, patient-, and study-level averages of pain intensity were added as predictor variables of completion rates on each of the 3 levels. Analyses were conducted using maximum likelihood parameter estimation in Mplus, version 8 [21]. Values of *P*<.05 were considered statistically significant.

### Power Calculations

Statistical power in multilevel models depends on several factors such as the sample sizes at each level of analysis and the intraclass correlations due to the clustering effect or observation dependence of lower-level units nested in higher-level units. We conducted a power analysis using Monte Carlo simulation [22] to determine the minimum effect sizes that would be detectable with 80% power (alpha=.05), given the sample sizes and intraclass correlations of the data analyzed in the present study (see Sample Size and Design Characteristics section below). The minimum detectable effect sizes were 0.04 at Level 1 and 0.1 at Level 2, corresponding with small effects following the Cohen [23] conventions. Owing to the limited sample size at Level 3 (the study level), the minimum detectable effect size at this level was 0.7, which was a large effect.

## Results

### Results of the Literature Search

**Figure 1:**
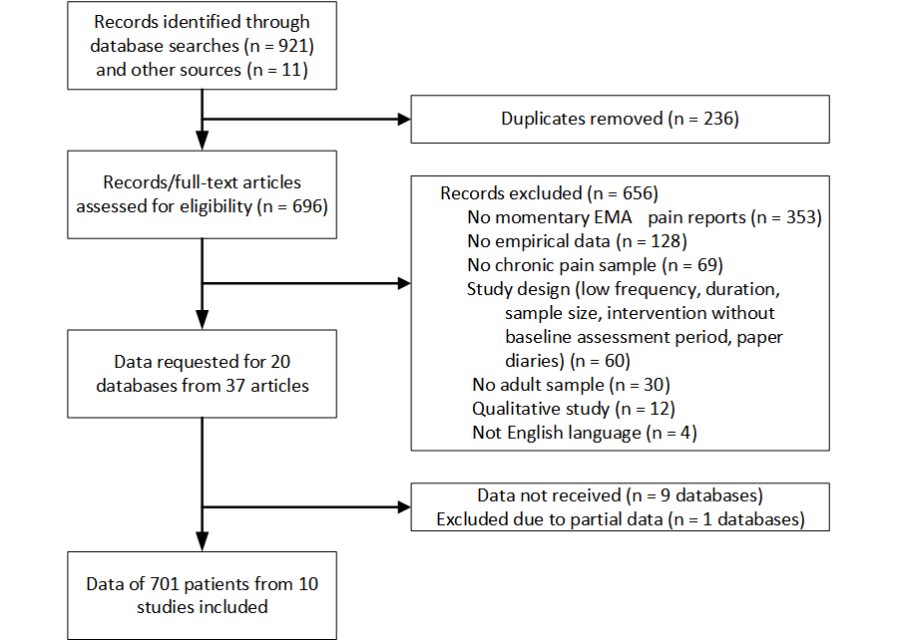
Flow diagram describing the identification of databases. EMA: ecological momentary assessment.

Our literature search identified 20 eligible databases from 37 articles (Figure 1). Authors of these articles were contacted by the research team. Original patient data were received for 10 of the 20 databases: Nine datasets were not received because the authors did not respond to the request or declined to provide the data or because the data were no longer available, and one dataset was not included because it provided only partial data without information on demographic predictor variables. One database consisted of three substudies comprising independent patient samples with different EMA sampling designs. These were separated into three datasets; thus, a total of 12 independent datasets were included in the analyses.

### Sample and Design Characteristics

Characteristics of the participants and studies are summarized in Table 1. Overall, our analyses included 7956 study days from a total of 701 patients. The study duration ranged from 4 to 28 days. The number of prompts per day ranged from 3 to 12 prompts, and the number of items per prompt ranged from 6 to 63 items.

### Descriptive Results of Ecological Momentary Assessment Completion Rates

**Figure 2:**
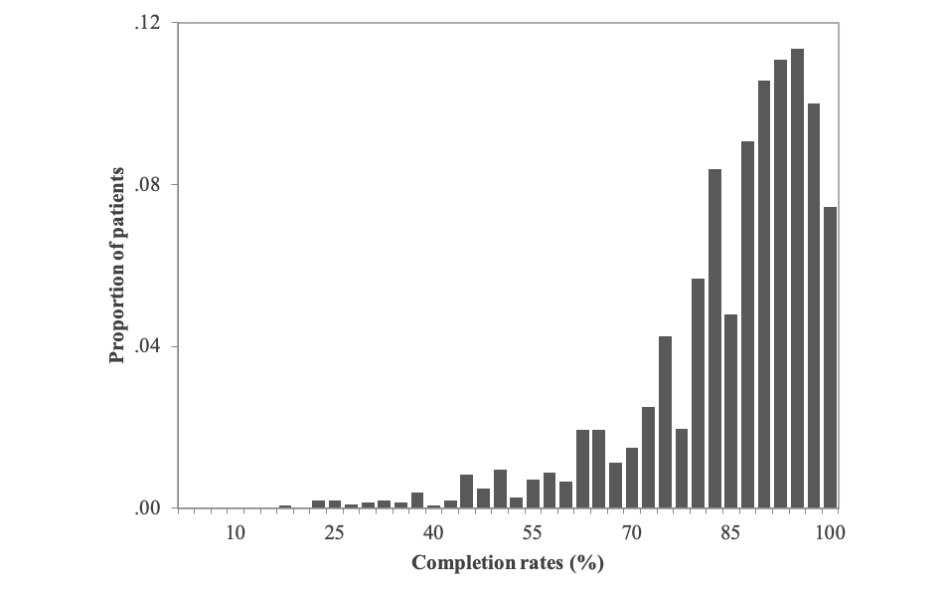
Distribution of patient-level average ecological momentary assessment completion rates.

The distribution of average daily completion rates by individuals is presented in Figure 2. The average completion rate was 85%, with daily completion rates <70% for 13% of the patients, <80% for 27% of the patients, and <90% for 60% of the patients. Initial multilevel models without predictor variables showed that 58% of the total variance in completion rates was attributable to within-person (day-to-day) variation: 26% to reliable differences between patients and 15% to differences among studies. Thus, investigation of predictors from different levels was warranted.

### Predictors of Ecological Momentary Assessment Completion Rates

**Figure 3:**
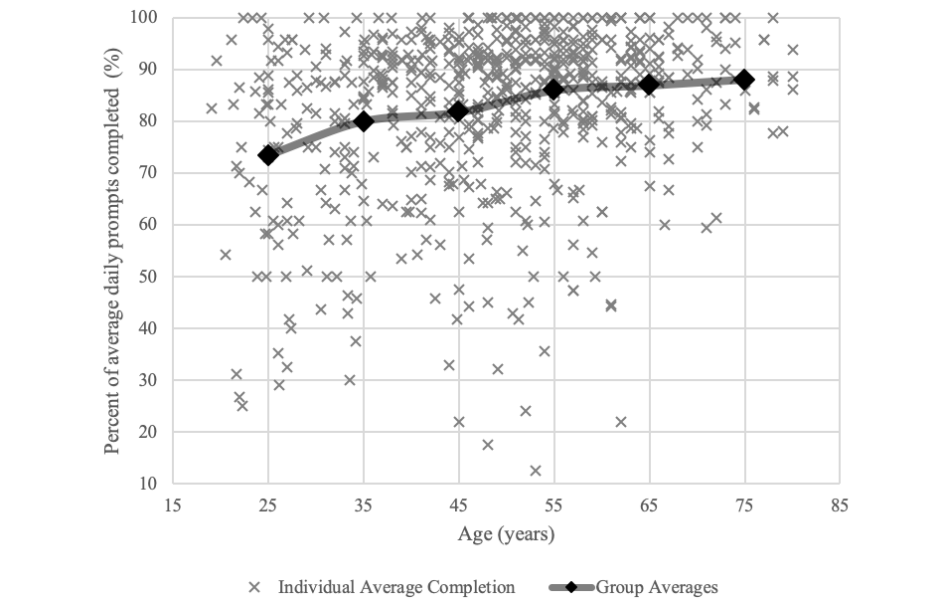
Scatter plot of average daily completion rates by age. An overlaying line graph represents average completion rates by patient age groups. For example, the average of the first group of patients in their 20s is indicated at the age of 25 years.

**Figure 4:**
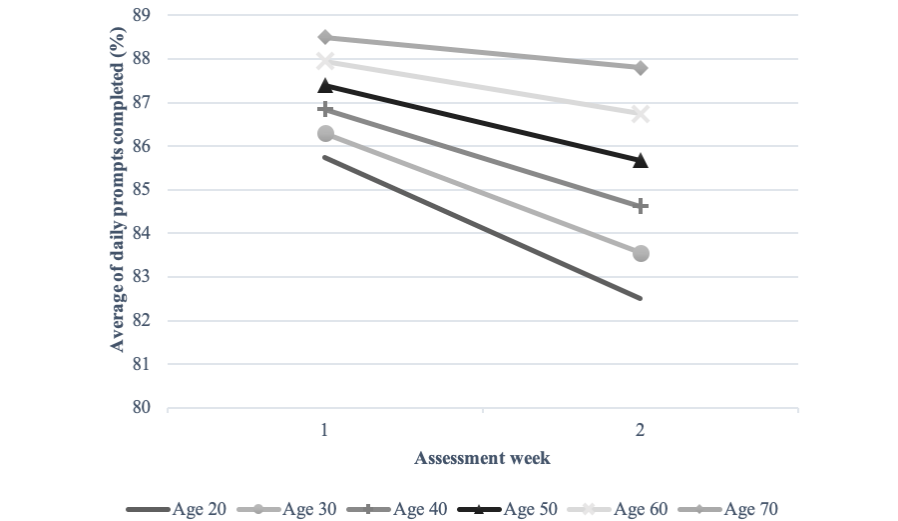
The cross-level interaction effect between age and study day.

Findings for the multilevel model predicting completion rates are summarized in Table 2. On the within-person level, we found a significant linear decline in daily completion over time (*b*=–2.29, *P*<.001); on average, completion rates decreased by approximately 2.0% per week of EMA sampling. On the between-person level, age showed a curvilinear relationship with completion rates (*b*_age_linear_=1.77, *P*=.002; *b*_age_quadratic_=–0.62, *P*=.009). Completion rates were highest among older patients (age≥60 years), and younger patients showed less completion (Figure 3). In addition, we found that the linear term of age significantly moderated the magnitude of changes in completion rates over time (*b*=0.56, *P*=.02). Younger patients had steeper declines over time as compared to older patients (Figure 4). Other patient characteristics—gender and chronic pain diagnosis—were not significant predictors in the model. On the between-study level, study duration, sampling density, and survey length were not significantly related to completion rates. Similarly, pain intensity levels were not significantly related to day-to-day variation in within-patient, between-patient, or study-level differences in completion rates.

**Table 1:** Participant and study level descriptive characteristics.

Characteristics			Statistics	Reference
**Participant level (n=701)**				
	Pain intensity, mean (SD), range		42.00 (20.54), 1.88-92.71	—^a^
	Age (years), mean (SD), range		48.70 (13.08), 19-80	—
	Female, n (%)		469 (67)	—
	**Diagnosis, n (%)**			
		Osteoarthritis	175 (25)	—
		Rheumatoid arthritis	71 (10)	—
		Fibromyalgia	73 (10)	—
		Mixed or others	386 (55)	—
**Study level (n=10), mean (SD), range**				
	Sample size		70.10 (22.75), 31-115	—
	**Study purpose, n (%)**			
		Within-person processes	5 (50)	[24-28]
		Intervention to reduce pain	1 (10)	[29]
		Methodological (eg, recall bias)	4 (40)	[30-33]
	**Study duration (days), mean (SD), range**		12.30 (8.71), 4-28	—
		4-7, n (%)	5 (50)	[24,28,29,31,33]
		8-14, n (%)	3 (30)	[25,26,32]
		15-28, n (%)	2 (20)	[27,30]
	**Sampling density (number of prompts per day)^b^, n (%)**		6.25 (2.60), 3-12	—
		3-5	5 (42)	[19,24,27,29,32]
		6-8	5 (42)	[25,26,30-32]
		9-12	2 (17)	[32,33]
	**Items per prompt, n (%)**		24.60 (18.93), 6-63	—
		6-10	2 (20)	[24,31]
		11-20	5 (50)	[25,29,30,32,33]
		21-63	3 (30)	[26-28]

^a^Not applicable.

^b^Values for sampling density are based on n=12 datasets (one study contained three datasets with different numbers of prompts by design).

**Table 2:** Three-level multilevel model of predictors of ecological momentary assessment completion.

Predictors				Estimate	Standard error	*P* value
**Fixed effects**						
	Intercept			88.61	16.26	<.001
	**Level 1 (day)**					
		Study days^a^		–2.29	0.32	<.001
		Daily average pain^b^		–0.23	0.20	.25
	**Level 2 (patient)**					
		**Age**				
			Linear	1.77	0.24	.002
			Quadratic^c^	–0.62	0.24	.009
		Female sex		–1.29	1.14	.26
		**Diagnosis**				
			Osteoarthritis	0.23	1.84	.90
			Rheumatoid arthritis	0.99	2.17	.65
			Fibromyalgia	0.64	1.76	.72
		Age×study days		0.56	0.23	.02
		Patient average pain^d^		0.03	0.24	.89
	**Level 3 (study)**					
		Duration (number of days)		0.26	0.36	.48
		Density (number of prompts per day)		–0.16	1.06	.88
		Lengths (number of items)		–0.17	0.16	.27
		Study average pain		–0.57	3.78	.88
**Random effects**						
	**Level 1 (day)**					
		Within-person residual		257.12	4.47	<.001
	**Level 2 (patient)**					
		Intercept		105.61	9.20	<.001
		**Slope**				
			Study day	3.39	1.56	.03
			Daily average pain	3.59	1.02	<.001
	**Level 3 (study)**					
		Intercept		58.46	25.09	.02
**Parameters**				23	—^e^	—
	–2log likelihood			67872.34	—	—
	AIC^f^			67918.35	—	—
	BIC^g^			68078.85	—	—

^a^Study day was coded in weekly units.

^b^Daily pain was within-person centered.

^c^Age was centered at 50 years.

^d^Patient-level pain was within-study centered.

^e^Not applicable.

^f^AIC: Akaike Information Criterion.

^g^BIC: Bayesian Information Criterion.

## Discussion

### Principal Findings

The goal of this study was to determine whether completion rates in EMA studies on chronic pain differ systematically according to characteristics of the situation, person, or EMA protocol. Analyzing data of 701 patients from 10 studies, we found an average EMA-completion rate of 85%. Although this rate may seem high, this finding is consistent with that of other studies on chronic pain [12,16]. The results revealed lower completion rates among younger patients than among older patients. In addition, we observed a decline in completion rates over the course of study protocols. The rate of this decline was moderated by respondent age: Younger patients showed a faster decline in completion rates than older patients.

### Overview

The effect of age may be a result of fewer competing demands from elderly patients in their daily lives due to, for example, retirement, as compared to younger patients. In fact, evidence suggests that younger respondents are more prone to inattention and carelessness when completing online surveys than older respondents [34]. Similarly, the observed decline in completion rates over time may be due to survey fatigue or loss of motivation over the study days. Okifuji et al [11] recommend limiting the period of EMA assessments to 1 week, but this limit may not be desirable when, for example, evaluating responses to changes in treatment or adjustment to new medications using EMA. Research is needed to identify feasible ways to ensure sustained patient engagement in EMA assessments over time including the use of monetary or motivational incentives, close participant-researcher interactions over the course of the study, and the use of emerging data-collection strategies aimed at reducing participant burden, such as “microinteraction-based” EMA (a method developed to answer few EMA items very quickly) [35] or “measurement burst” designs (multiple brief EMA periods repeated over time) [36].

Our analyses did not support several *a priori* hypotheses. We did not detect any gender differences in the EMA-completion rates. In fact, previous findings of lower completion rates among men were primarily based on healthy samples [6-8], and studies on chronic pain did not identify such a pattern [10,11]. In addition, we found no evidence that either day-to-day variations in pain intensity or differences in pain intensity levels between participants or studies were systematically associated with EMA completion. This finding is especially important because an association between high pain levels and a low likelihood of responding to EMA surveys could severely undermine the validity of the EMA data collected to monitor patients’ pain in everyday life. An important caveat of our analyses is that they were necessarily based on averages of pain from EMA prompts that were not missed by patients; considering that pain levels for missed EMA prompts are not known, the possibility that EMA prompts are more likely missed when patients are in higher (or lower) pain at the time of the prompt cannot be excluded. Our findings may have also resulted from the salience of pain experiences in the study samples. Participation in research wherein pain is of immediate relevance and intrinsic importance might have contributed to patients’ motivation to complete the momentary assessments even in times of high pain intensity. This perspective aligns with our result of no differences in the EMA-completion rates according to the chronic pain diagnosis. The characteristics of the chronic pain experience can vary substantially between diagnoses; for example, high levels of fatigue and cognitive difficulties are more strongly associated with some diagnoses than with other pain diagnoses, and this might have contributed to differences in completion rates. However, the present results suggest that more complex symptomatology per se might not preclude patients’ engagement in the momentary assessments.

At the between-study level, we did not detect any associations between design features related to participant burden (overall length of a given study, sampling density, and number of EMA survey items) and completion rates, which was surprising. Given the intensive nature of EMA protocols, participant burden has often been viewed as a major factor contributing to noncompliance [7,12]. One possibility is that other study design factors (eg, frequent contacts with participants to keep them motivated) are more important than load for continued engagement in EMA protocols, and these factors should be studied in future research. Additionally, despite the sizeable number of participants included in our analyses, IPD meta-analysis can be negatively influenced by a low statistical power at the highest (between-study) level of analysis, which may have limited our ability to detect effects based on study-level design features [37].

### Limitations and Future Directions

A limitation of our study is the potential selectivity bias: We were able to include 10 of 20 eligible datasets. Inclusion of >90% of the eligible studies in IPD meta-analyses has been suggested as an ideal target [38], although, in practice, many IPD meta-analyses include <80% of the eligible datasets [39]. To evaluate the potential for selection bias in the data available for the present analyses, we examined the pooled average completion rate reported for eligible studies that were not included in the analyses; of 10 studies, 7 provided average completion rates in the published reports. The weighted average completion rate in these studies was 78.2%, suggesting a potential upward bias of completion rates in the data that were available for our analyses.

When calculating completion rates, we relied on a fixed number of EMA prompts, unless studies employed a variable prompting schedule based on patients’ waking hours. As such, we assumed that each participant in those studies consistently received the same number of prompts. However, this assumption may have sometimes been violated due to the potential of malfunctioning of data-collection devices or limitations in their configuration capability (ie, prompting during sleep). Thus, some of the calculated daily completion rates may have underestimated participants’ actual completion rates.

Our study is also limited by the number and types of predictor variables that were consistently available across the different datasets. Data on additional predictors such as negative affect, disability status, and stress levels were not consistently available but are undoubtedly candidates for understanding EMA completion in chronic pain. Similarly, EMA data-capturing methods differ among many intricate dimensions that could be theoretically important predictors of EMA completion rates, including whether participants should be allowed to delay the assessment (ability to “snooze” or “suspend” an assessment), how fast a participant is expected to start an assessment (ie, the time window during which an assessment stays open for completion), the frequency of contact between the research team and the participant, the availability of reminders to complete the assessment (and the type and frequency of reminders), and the expectation of individualized feedback at the end of the study. Finally, our findings may not be generalizable to patients with other illnesses or healthy populations.

### Conclusions

In summary, our IPD meta-analysis showed no evidence to suggest that EMA-completion rates in chronic pain differ by medical diagnoses; gender; EMA study design features related to participant burden; or variations in pain levels across days, patients, or studies. These findings support the use of EMA data-collection methods for careful assessment of patients’ pain and other experiences. Future EMA research in chronic pain should note that study length and young age can affect the quality of the momentary data and devise strategies to maximize EMA-completion rates across different age groups and study days.

## Abbreviations

AIC: Akaike information criterion
BIC: Bayesian information criterion
EMA: ecological momentary assessment
IPD: individual patient data
MAR: missing at random

## References

[1] Shiffman S, Stone AA, Hufford MR (2008). Ecological momentary assessment. Annu Rev Clin Psychol.

[2] Hamaker EL, Wichers M (2017). No Time Like the Present: Discovering the Hidden Dynamics in Intensive Longitudinal Data. Curr Dir Psychol Sci.

[3] Stone AA, Shiffman S (2002). Capturing momentary, self-report data: a proposal for reporting guidelines. Ann Behav Med.

[4] Courvoisier DS, Eid M, Lischetzke T (2012). Compliance to a cell phone-based ecological momentary assessment study: the effect of time and personality characteristics. Psychol Assess.

[5] Schafer JL, Graham JW (2002). Missing data: Our view of the state of the art. Psychol Methods.

[6] Messiah A, Grondin O, Encrenaz G (2011). Factors associated with missing data in an experience sampling investigation of substance use determinants. Drug Alcohol Depend.

[7] Sokolovsky AW, Mermelstein RJ, Hedeker D (2014). Factors predicting compliance to ecological momentary assessment among adolescent smokers. Nicotine Tob Res.

[8] Silvia PJ, Kwapil TR, Eddington KM, Brown LH (2013). Missed beeps and missing data: Dispositional and situational predictors of nonresponse in experience sampling research. Soc Sci Comput Rev.

[9] McLean DC, Nakamura J, Csikszentmihalyi M (2017). Explaining System Missing. Social Psychological and Personality Science.

[10] Aaron LA, Mancl L, Turner JA, Sawchuk CN, Klein KM (2004). Reasons for missing interviews in the daily electronic assessment of pain, mood, and stress. Pain.

[11] Okifuji A, Bradshaw DH, Donaldson GW, Turk DC (2011). Sequential analyses of daily symptoms in women with fibromyalgia syndrome. J Pain.

[12] Morren M, van Dulmen S, Ouwerkerk J, Bensing J (2009). Compliance with momentary pain measurement using electronic diaries: A systematic review. Eur J Pain.

[13] Riley RD, Lambert PC, Abo-Zaid G (2010). Meta-analysis of individual participant data: rationale, conduct, and reporting. BMJ.

[14] Curran PJ, Hussong AM (2009). Integrative data analysis: The simultaneous analysis of multiple data sets. Psychol Methods.

[15] Hussong AM, Curran PJ, Bauer DJ (2013). Integrative data analysis in clinical psychology research. Annu Rev Clin Psychol.

[16] May M, Junghaenel DU, Ono M, Stone AA, Schneider S (2018). Ecological Momentary Assessment Methodology in Chronic Pain Research: A Systematic Review. J Pain.

[17] Broderick JE, Schwartz JE, Shiffman S, Hufford MR, Stone AA (2003). Signaling does not adequately improve diary compliance. Ann Behav Med.

[18] Stone AA, Shiffman S, Schwartz JE, Broderick JE, Hufford MR (2002). Patient non-compliance with paper diaries. BMJ.

[19] Heron KE, Smyth JM (2010). Ecological momentary interventions: Incorporating mobile technology into psychosocial and health behaviour treatments. Brit J Health Psych.

[20] Warton DI, Hui FKC (2011). The arcsine is asinine: The analysis of proportions in ecology. Ecology.

[21] Muthén LK, Muthén BO (1998). Mplus user's guide, 8th edition.

[22] Muthén LK, Muthén BO (2002). How to Use a Monte Carlo Study to Decide on Sample Size and Determine Power. Structural Equation Modeling: A Multidisciplinary Journal.

[23] Cohen J (1988). Statistical power analysis for the behavioral sciences.

[24] Bruehl S, Liu X, Burns JW, Chont M, Jamison RN (2012). Associations between daily chronic pain intensity, daily anger expression, and trait anger expressiveness: an ecological momentary assessment study. Pain.

[25] Viane I, Crombez G, Eccleston C, Devulder J, De Corte W (2004). Acceptance of the unpleasant reality of chronic pain: effects upon attention to pain and engagement with daily activities. Pain.

[26] Huijnen IPJ, Verbunt JA, Roelofs J, Goossens M, Peters M (2009). The disabling role of fluctuations in physical activity in patients with chronic low back pain. Eur J Pain.

[27] Peters ML, Sorbi MJ, Kruise DA, Kerssens JJ, Verhaak PF, Bensing JM (2000). Electronic diary assessment of pain, disability and psychological adaptation in patients differing in duration of pain. Pain.

[28] Smyth JM, Zawadzki MJ, Santuzzi AM, Filipkowski KB (2014). Examining the effects of perceived social support on momentary mood and symptom reports in asthma and arthritis patients. Psychol Health.

[29] Litt MD, Shafer DM, Ibanez CR, Kreutzer DL, Tawfik-Yonkers Z (2009). Momentary pain and coping in temporomandibular disorder pain: exploring mechanisms of cognitive behavioral treatment for chronic pain. Pain.

[30] Broderick JE, Schwartz JE, Vikingstad G, Pribbernow M, Grossman S, Stone AA (2008). The accuracy of pain and fatigue items across different reporting periods. Pain.

[31] Smith DM, Parmelee PA (2016). Within-day variability of fatigue and pain among African Americans and non-Hispanic Whites with osteoarthritis of the knee. Arthritis Care Res.

[32] Stone AA, Broderick JE, Schwartz JE, Shiffman S, Litcher-Kelly L, Calvanese P (2003). Intensive momentary reporting of pain with an electronic diary: Reactivity, compliance, and patient satisfaction. Pain.

[33] Stone AA, Broderick JE, Schneider S, Schwartz JE (2012). Expanding options for developing outcome measures from momentary assessment data. Psychosom Med.

[34] Schneider S, May M, Stone AA (2017). Careless responding in internet-based quality of life assessments. Qual Life Res.

[35] Intille S, Haynes C, Maniar D, Ponnada A, Manjourides J (2016). μEMA: Microinteraction-based Ecological Momentary Assessment (EMA) Using a Smartwatch. Proc ACM Int Conf Ubiquitous Comput.

[36] Sliwinski M (2008). Measurement-Burst Designs for Social Health Research. Social Pers Psych Compass.

[37] Hedges LV, Pigott TD (2004). The power of statistical tests for moderators in meta-analysis. Psychol Methods.

[38] Sterne JAC, Egger M, Moher D (2008). Addressing reporting biases. Cochrane Handbook for Systematic Reviews of Interventions.

[39] Ahmed I, Sutton AJ, Riley RD (2012). Assessment of publication bias, selection bias, and unavailable data in meta-analyses using individual participant data: a database survey. BMJ.

